# Substance Use Disorders and the Psychosis Spectrum: Assessment, Clinical Challenges and Management

**DOI:** 10.3390/jcm15041562

**Published:** 2026-02-16

**Authors:** Thomas Paparrigopoulos, Eleftherios Mellos, Charidimos Tzagarakis

**Affiliations:** 1 School of Medicine, National and Kapodistrian University of Athens, 11528 Athens, Greece; 2 National Organization for Prevention and Addiction Treatment (NOPAT), 10433 Athens, Greece; elmellos1962@gmail.com; 3 Department of Psychiatry, Medical School, University of Crete, 70013 Heraklion, Greece; haristz@uoc.gr

**Keywords:** substance use disorders, substance-induced psychosis, psychosis, schizophrenia, assessment, diagnosis, clinical symptoms, pathophysiology, management, treatment

## Abstract

The intricate relationship between substance-induced psychotic disorders and primary disorders of the psychosis spectrum requires practitioners to meticulously evaluate patient history, substance use patterns, and clinical symptomatology. Also, the dual diagnosis of SUDs and psychotic disorders exacerbates treatment challenges, often leading to the need to coordinate differing treatment modalities and fragmented care. A nuanced understanding of the interplay between SUDs and the psychosis spectrum is crucial for effective assessment and comprehensive management strategies. In this review, the unique challenges presented by this complex clinical population are examined, and management strategies are explored.

## 1. Introduction

The latest release of the EUDA European Drug Report 2025, together with the Monitoring the Future Panel Study and the UNODC World Drug Report 2025, highlights that drug use across the globe is not only widespread but also increasingly diverse, potent, and unpredictable [1,2,3].

The EUDA report documents a sharp rise in the availability of cocaine, ketamine and novel synthetic drugs—including synthetic stimulants, semi-synthetic cannabinoids, and new psychoactive substances—often sold in mixed or adulterated forms. Data also highlight growing polysubstance use, which complicates patterns of intoxication and harm [1]. Meanwhile, the Monitoring the Future Panel Study confirms that drug use behaviors continue well into adulthood in the United States, underscoring that substance exposure—including cannabis, psychedelics, and other drugs—is not confined to adolescence but persists across life stages [2]. At the global scale, the UNODC report shows that an estimated 316 million people used at least one illicit drug in 2023—about 6% of the population aged 15–64—up from 5.2% a decade earlier [3].

The emergence of novel psychoactive substances (NPS) and the dynamic evolution of illicit drug markets pose significant challenges for the clinical assessment of substance-related psychosis. These compounds, including, as stated above, synthetic cannabinoids, novel stimulants, and designer psychedelics, exhibit unpredictable pharmacological properties, high potency, and limited clinical evidence, complicating the differentiation between substance-induced psychosis and primary psychotic disorders. Furthermore, standard toxicological screens frequently fail to detect NPS, increasing the risk of diagnostic misclassification (see below). These limitations underscore the need for comprehensive clinical evaluation, heightened awareness of emerging substances, and improved toxicological detection methods [4].

Taken together, these findings signal a critical public health alarm, because as drug markets diversify and intensify, individuals face ever-greater exposure not only to established risks but also to novel, potent, and unpredictable combinations that considerably raise the potential for substance-induced psychosis and long-term psychiatric morbidity.

Psychosis, broadly defined, refers to a disconnection from reality characterized by symptoms such as hallucinations, delusions, and impaired thought processes. While psychosis is most associated with primary psychiatric disorders like schizophrenia, it can also emerge secondary to substance use. Substance-induced psychosis (SIP) occurs when these symptoms arise during or shortly after the use of, or withdrawal from, psychoactive substances such as cannabis, amphetamines, cocaine, hallucinogens, alcohol, or synthetic drugs [5,6]. Unlike primary psychotic disorders, SIP is traditionally considered temporary, with symptoms subsiding after the drug has been metabolized or the withdrawal process is complete. However, growing evidence suggests that in a substantial subset of individuals, SIP serves as a prodromal or precipitating factor in the development of persistent psychotic conditions [7]. This blurred boundary between SIP and primary psychosis presents diagnostic and clinical challenges, warranting a nuanced understanding of the phenomenon.

## 2. Methods

This article presents a narrative review of literature examining the relationship between substance use disorders and the psychosis spectrum. A literature search was conducted in the electronic databases PubMed and Scopus, using keywords such as “substance use disorders”; “substance-induced psychosis”; “psychosis”; “schizophrenia”; “assessment”; “diagnosis”; “clinical symptoms”; “pathophysiology”; “management”; “treatment”. The search strategy did not follow a strictly systematic or fully reproducible protocol. Articles were selected based on their relevance to the topic, scientific quality, and clinical significance, with particular emphasis on studies investigating etiological mechanisms, clinical characteristics, and therapeutic approaches.

## 3. Epidemiology

The prevalence and incidence of substance-induced psychosis vary by population, geographic region, and substance type. In a Norwegian catchment area study, the treated incidence for SIP was found to be 6.5/100,000 persons per year [6]. Among individuals presenting with drug intoxication in the context of the Euro-DEN project, there was a prevalence of psychosis of 6.3% [8]. A Canadian study of the transition to schizophrenia among people who attend emergency departments due to substance use showed an increased risk of transition regardless of whether there was psychosis present at presentation, although the latter resulted in a higher risk (163.2 times higher than the general population vs. 9.8 times higher) [9]. Cannabis is the most frequently implicated substance in cases of SIP. Its increasing availability, legalization in various countries, and rising potency of its active compounds (notably THC) have contributed to a growing number of psychotic episodes linked to its use. Amphetamines and other stimulants also represent significant contributors, especially in regions with high methamphetamine use. Cocaine, synthetic cannabinoids (e.g., “spice”), alcohol, hallucinogens (e.g., LSD and psilocybin), and opioids also account for a significant share of cases [8]. Age and gender are notable demographic factors; young males, particularly those in their teens or early twenties, are at heightened risk of developing SIP. This demographic corresponds with peak periods of substance experimentation and recreational use, aligning temporally with the onset of many psychiatric disorders [6]. Finally, the prevalence of Alcohol-Induced Psychotic Disorder (AIPD, see below) varies widely. In Germany, the annual prevalence of alcohol-induced psychotic disorders and delirium was 0.6–0.7% and 4.9–7.4%, respectively [10,11], while in a Finnish study that showed it was more common among men of working age, the general population lifetime prevalence was 0.41%, or 4% for people with alcohol dependence syndrome [12].

## 4. Assessment, Differential Diagnosis

From a nosological perspective, the distinction between SIP and primary psychosis is essential but often ambiguous. The need to differentiate transient, substance-triggered psychosis from the first manifestation of a chronic disorder underscores the importance of comprehensive assessment, longitudinal observation, and the integration of clinical, toxicological, and psychosocial data. In addition, clinicians must differentiate between psychotic symptoms associated with substance use and appearing in clear sensorium and those attributable to withdrawal syndromes, intoxication, or delirium. Structured follow-up assessments at regular intervals are needed to monitor relapses, conversion, or functional outcomes.

Key differentiators include the timing of symptom onset relative to substance use, the presence of prior psychiatric history, the pattern of symptom persistence during abstinence, and family history of mental illness [13]. SIP tends to present abruptly and is temporally associated with substance ingestion or withdrawal. Indeed, diagnostic frameworks such as the DSM-5-TR and ICD-11 emphasize the temporal relationship between substance exposure and symptom onset, and relevant clinical guidelines emphasize the importance of establishing a temporal link between substance use and the onset of psychotic symptoms accordingly, yet they also acknowledge the diagnostic uncertainty that arises when psychotic symptoms persist beyond expected intoxication or withdrawal periods [14,15,16]. The individual typically maintains insight into the unusual nature of their experiences [13], especially as the acute effects of the substance wear off. In contrast, primary psychotic disorders often emerge insidiously, with a prodromal period of subthreshold symptoms and functional decline. Mental status examinations, physical assessments, and laboratory tests (including urine drug screens and blood alcohol levels) are important components of the diagnostic process. Mental status examination assists in differentiating psychosis from delirium, which may present with fluctuating attention, disorientation, and perceptual misinterpretations rather than structured delusions or hallucinations. Also, physical examination should evaluate for signs of intoxication, autonomic instability, or neurological symptoms. Certain substances produce distinctive physical findings—such as mydriasis, hyperthermia, or hypertension with stimulant use—which may aid in diagnosis.

Longitudinal observation is often necessary. If symptoms persist beyond four weeks of abstinence, clinicians may need to revise the diagnosis. Additionally, patients with SIP typically do not show the cognitive impairments and social deterioration characteristic of schizophrenia, though these differences can become less apparent with repeated episodes. Family history also plays an essential role. Individuals with relatives affected by schizophrenia or other psychoses are more likely to transition from SIP to a primary psychotic disorder, suggesting a shared vulnerability. On the other hand, a recent health register-based study showed an increased incidence of self-harm/suicidality events as well as of ADHD in patients with first-episode SIP versus primary psychosis [17]. Finally, for AIPD, differentiating elements from primary psychosis are the older age of onset as well as the virtual absence of thought disorder, disorganized and negative symptoms, and the different family histories [18].

Furthermore, symptoms should be consistent with the known psychotomimetic profile of the substance used. Special attention should be given to polysubstance use, as concurrent ingestion of multiple agents (e.g., cannabis and stimulants) can produce synergistic psychotomimetic effects or confound interpretation of laboratory findings.

Finally, as indicated above, toxicological screening is essential but must be interpreted cautiously. Standard urine or blood assays can detect common substances such as cannabinoids, cocaine, amphetamines, and opiates but may fail to identify newer synthetic compounds (e.g., synthetic cannabinoids, cathinones, or hallucinogens) that require specialized chromatographic or mass spectrometric methods [19].

Timely sampling is critical, as detection windows vary significantly [20] by substance, dose, and individual metabolism (e.g., hours for LSD, days for cocaine, weeks for cannabis). Repeated testing may be warranted in cases of diagnostic uncertainty or suspected ongoing use.

Common pitfalls in diagnosing SIP include: (a) Attributing primary psychosis to substance use based solely on a positive toxicology result; (b) Failing to recognize that certain substances (e.g., cannabis, hallucinogens) may unmask latent psychotic vulnerability; and (c) Overlooking the contribution of medical or neurological comorbidities.

Several structured diagnostic tools have been developed to assist clinicians, including the Psychiatric Research Interview for Substance and Mental Disorders (PRISM) [21] and the Addiction Severity Index [22]. However, these tools either require specialized training and may be time-consuming, making them less feasible in many real-world clinical environments, or they may not be reliable for patients with the most severe psychopathology.

## 5. Symptoms and Clinical Characteristics

The symptomatology of SIP closely resembles that of primary psychotic disorders, particularly schizophrenia. Most commonly, SIP presents positive psychotic symptoms including auditory and visual hallucinations, persecutory delusions, paranoid ideation, and disorganized speech or behavior. Disorientation, agitation, and intense fear or suspicion are frequent accompaniments. Although traditionally, negative symptoms such as flattened affect, social withdrawal, and avolition are generally considered less prominent or absent in SIP, this is far from pathognomonic [23], and in fact, NMDA antagonist-induced psychosis (PCP/Ketamine) and solvent-induced psychosis may often present with negative symptomatology [24]. Mood symptoms, including anxiety and depression, often co-occur; for instance, cannabis-induced psychosis is frequently associated with paranoid dysphoria [25]. Cocaine- and amphetamine-related psychosis may also present with heightened paranoia [26], as well as frequent visual hallucinations [27]. The clinical presentation can vary significantly depending on the substance involved (see Table 1)

***Cannabis:*** hallucinations (mostly auditory), paranoia, and emotional lability, occasionally negative symptoms [25].

***Amphetamines:*** paranoid delusions, tactile hallucinations (e.g., “formication”), agitation, and impaired reality testing [28].

***Cocaine:*** persecutory delusions, ideas of reference, heightened suspiciousness, and multimodal hallucinations, with auditory and tactile hallucinations being the most common. “Cocaine bugs” or formication are particularly characteristic [29].

***Phencyclidine (PCP)/Ketamine:*** severe agitation, paranoia, thought disorder, hallucinations (both auditory and visual), and delusional beliefs. Behavioral disturbances—including disorganization, aggression, and catatonic-like states—are not uncommon. Compared with ketamine, PCP is more likely to produce prolonged or recurrent psychotic symptoms, likely due to its higher potency and slower elimination. Ketamine, at sub-anesthetic doses, induces dissociation, perceptual distortions, depersonalization, and transient psychosis-like phenomena. These symptoms typically resolve within hours, but high doses or chronic use can result in more persistent disturbances [30].

***Psychedelics (LSD, psilocybin, mescaline, DMT):*** visual distortions, alterations in sensory processing and belief formation, depersonalization, derealization [31]; psychedelic experiences, by contrast to primary psychoses, are usually time-limited and recognized by users as drug-induced.

***Solvents:*** an “amotivational syndrome” often characterizes these psychoses [24], and the inhalation of certain volatiles in adolescence is often accompanied by the emergence of auditory hallucinations [32].

***Synthetic Cathinones (such as Mephedrone):*** an “agitated psychosis” syndrome may present with acute persecutory delusions and auditory, visual, and tactile hallucinations with marked disorganization and violent behavior [33]; see Box 1.

Box 1 **What are synthetic Cathinones?**

**Synthetic Cathinones:** Also known as “Bath Salts”, these are synthetic variations on the Cathinone substances derived from the plant “Catha Edulis” (khat plant), widely present in Eastern Africa and the Arabian Peninsula. The leaves of the plant are chewed to give a stimulant effect. Synthetic Cathinones are relatively easily modifiable to circumvent chemical detection and have amphetamine-like effects that include psychotic symptoms such as hallucinations and paranoia.

***Synthetic cannabinoids:*** severe agitation, confusion, bizarre behavior, psychosis (paranoia, persecutory delusions, auditory or visual hallucinations, disorganized thought processes), and depersonalization or dissociation that may be prolonged. Most episodes occur shortly after substance use or during withdrawal. In many cases, symptoms are resolved within days to weeks of abstinence, but in others they persist, especially with repeated exposure [8,34,35]; see Box 2.

Box 2 **What are synthetic and semi-synthetic cannabinoids?**

**Synthetic and semi-synthetic cannabinoids:** These are a diverse group of laboratory-produced compounds (including but not limited to Spice, JWH-018 and K2) designed to activate cannabinoid receptors, primarily CB1, with far greater potency and efficacy than Δ9-tetrahydrocannabinol (THC) but low cannabidiol (CBD) type of activity. These substances are often sprayed onto plant material and sold as legal alternatives to cannabis. However, their pharmacological unpredictability and high receptor affinity present significant risks, including a strong association with acute and persistent psychotic symptoms [34]. The unregulated nature of synthetic cannabinoid products—often containing rapidly changing chemical formulations—further complicates risk assessment, as users may unknowingly ingest substances with extreme potency.

***Alcohol:*** psychotic symptoms can occur in several clinical conditions related to alcohol abuse, such as intoxication, withdrawal, alcohol-induced psychotic disorder (AIPD), and delirium. The terms “alcoholic hallucinosis” and “alcohol-induced psychotic disorder” (AIPD) are often used alternatively, with the former being older and more descriptive [12]. AIPD can emerge right after consuming large amounts of alcohol and is not necessarily linked to how long alcohol dependence has been present [36].

## 6. Neurobiological Mechanisms and Pathophysiology

Substance-induced psychoses associated with cannabis, hallucinogens, and amphetamines have a substantial risk of transition to schizophrenia and should be a focus for assertive psychiatric intervention [7]. The diverse pharmacological profiles of substances of abuse known to induce psychosis imply that multiple neurophysiological mechanisms can underlie its onset. Below, we outline some of the key relevant frameworks (see Table 1):

**Amphetamines** can result in both an increase in dopamine in the synaptic cleft through reuptake inhibition and pre-synaptic transporter reversal, as well as an increase in intracellular dopamine with associated cytotoxicity. There is also an increase in norepinephrine, again through reuptake inhibition [37], which is also thought to contribute to the potential development of psychosis [38]. Symptoms of amphetamine psychosis can, on occasion, appear as flashbacks of the initial psychotic experience [23]. Methamphetamine is a particularly insidious substance due to its high lipophilicity, which allows it to readily cross the blood–brain barrier. Its availability in highly potent forms, such as crystal meth, combined with active and increasingly frequent use, substantially elevates the risk of psychosis [28]. Amphetamine-induced psychosis has been typically associated with positive symptomatology [39].

**3,4-methylenedioxymethamphetamine (MDMA)**, commonly known as “ecstasy” or “Molly”, is a synthetic psychoactive substance with stimulant and entactogenic properties. Accumulating evidence indicates that particularly high-dose, frequent, or adulterated use can be associated with psychotic symptoms in vulnerable individuals. MDMA primarily increases extracellular serotonin, dopamine, and norepinephrine through reversal of monoamine transporters. The intense serotonergic surge can acutely alter perception, cognition, and mood, which may progress to transient psychotic symptoms in susceptible individuals, especially in overstimulating environments. Dopaminergic release—although less pronounced than that of amphetamines—may contribute to psychosis risk, particularly when MDMA is consumed at high purity or combined with other stimulants. Sleep deprivation, dehydration, hyperthermia, and polysubstance use—all common in recreational MDMA settings—further amplify psychosis risk [40].

**Cocaine**-induced paranoia is frequent [26], and a cocaine-induced psychotic syndrome with positive symptom preponderance can occur. The underlying neurobiological mechanism appears to primarily involve increased inhibition of dopamine reuptake [41], particularly through dysregulation of D2 receptor activity within the mesolimbic system [42]. Functional imaging studies demonstrate heightened activation in limbic regions during intoxication, while chronic use may lead to long-term alterations in dopaminergic receptor expression, glutamatergic signaling, and stress-related neurocircuitry [43,44,45,46].

**Synthetic Cathinones** are also known to easily cross the blood–brain barrier, interact and cause the loss of dopamine transporters and generally inhibit 5HT, norepinephrine, and DA reuptake [33,35]. These mechanisms produce substantial elevations in extracellular dopamine and norepinephrine, leading to increased arousal, euphoria, and locomotor activity.

**Phencyclidine (PCP)/Ketamine** are dissociative anesthetics that act as NMDA (glutamate) non-competitive receptor antagonists, which bind at a specific NMDA receptor site, as well as partial D2 agonists [47]. NMDA receptor blockade disrupts excitatory–inhibitory balance in cortical circuits, particularly by inhibiting glutamatergic drive onto GABAergic interneurons. This disinhibition results in increased cortical glutamate release and downstream dopaminergic dysregulation in mesolimbic and mesocortical pathways [30]. Their effect on increasing glutamate and dopamine neurotransmission is thought to be responsible for drug-induced psychosis, which, unlike amphetamine and generally stimulant-induced psychosis, can also present with negative symptomatology.

**Psychedelic drugs,** such as lysergic acid diethylamide (LSD), psilocybin, mescaline, and N,N-dimethyltryptamine (DMT), are a class of substances that primarily exert their effects through agonism or partial agonism at the serotonin 5-HT2A receptor. This activity leads to profound alterations in perception, cognition, and sense of self. **LSD** has an agonist effect on serotonin 5-HT2A autoreceptors in the raphe nucleus projections to the thalamus, which in turn seems to result in increased glutamatergic activity in cortical and subcortical areas [47]. **Psilocybin** modulation of 5-HT2A and 5-HT1A receptors is thought to be involved in visual hallucinations [48]. Neurobiologically, the intense sensory distortions and thought disturbances are linked to disrupted thalamocortical signaling and destabilization of hierarchical predictive processing, leading to a breakdown in the brain’s usual filtering of sensory information [49]. In recent years, psychedelics have been investigated for potential therapeutic value in conditions such as depression, anxiety, and post-traumatic stress disorder [50]. However, concerns persist regarding the precipitation of sustained psychotic and/or mood episodes, particularly in individuals with underlying neurobiological vulnerabilities [51,52].

**Cannabis:** A complex and still not well-understood mechanism seems to be related to acute psychotic symptomatology after cannabis use, with the responsible compound being Δ9-tetrahydrocannabinol (THC). The risk of developing psychotic symptoms is dose-dependent, with daily or near-daily use conferring the highest vulnerability. The elevated concentration of Δ9-tetrahydrocannabinol (THC) in modern cannabis products appears to be a critical factor. THC is known to dysregulate dopaminergic signaling, particularly in mesolimbic pathways implicated in psychosis. Conversely, cannabidiol (CBD), another major cannabinoid, may have antipsychotic or protective effects; thus, the THC:CBD ratio is increasingly recognized as important in modulating risk [53,54]. Endocannabinoid receptors, particularly CB1 receptors (CB1-R), are implicated in these processes, along with a possible contribution from GABA-deficient states [55]. One proposed model suggests that exogenous cannabinoids disrupt pyramidal cell activity through interactions with CB1-R, which are widely expressed in both the cerebral cortex and hippocampus [56].

Activation of CB1 receptors by Δ9-tetrahydrocannabinol (THC) modulates glutamatergic and GABAergic neurotransmission, producing acute effects such as altered perception and impaired cognition. In susceptible individuals, repeated exposure may lead to persistent dysregulation of neural circuits involved in salience processing, a core feature of psychotic disorders. Functional neuroimaging studies indicate that individuals who use cannabis and present with psychosis exhibit altered connectivity within fronto-striatal and default-mode networks compared with non-using patients, suggesting interactive effects between cannabis exposure and underlying neural vulnerability [57]. The causal role of cannabis in psychosis remains debated, as confounding factors may contribute. Individuals with a predisposition to psychosis—due to genetic vulnerability, childhood trauma, or pre-existing subclinical symptoms—are more likely to engage in heavy cannabis use. However, twin and longitudinal studies suggest that cannabis use independently predicts subsequent psychotic outcomes, even after controlling for these vulnerabilities. The strongest evidence indicates that cannabis can precipitate psychosis in individuals at elevated risk and may accelerate the transition from a prodromal phase to a first psychotic episode [58].

**Synthetic cannabinoids (SCs)** are full agonists at cannabinoid receptors and bind with higher affinity than Δ9-tetrahydrocannabinol (THC), which acts as a partial agonist. Moreover, the potentially protective effects of cannabidiol (CBD) against psychosis are absent in SC preparations [35]. These differences result in markedly amplified downstream signaling. Activation of CB1 receptors modulates γ-aminobutyric acid (GABA) and glutamate release, thereby affecting neural circuits involved in reward, memory, and perception. Excessive CB1 stimulation can destabilize dopaminergic regulation within mesolimbic pathways, creating neurochemical conditions conducive to psychosis [59]. In addition, many SCs have longer half-lives and generate unpredictable metabolic by-products, further increasing their psychiatric toxicity [60].

**Alcohol:** Evidence from experimental animal models suggests dysregulation of the glutamate–GABA–glutamine cycle, characterized by increased GABA and glutamine levels in the cingulate cortex and reduced GABA concentrations in the nucleus accumbens [61]. Alcohol exposure may also be associated with increased dopamine release, as observed in severe alcohol withdrawal states that are frequently accompanied by psychotic symptoms [62]. In alcohol-induced psychotic disorder (AIPD), there is a notable absence of formal thought disorder and negative symptomatology [63].

**Solvents:** The mechanisms of action of solvent exposure also appear to involve disruption of the GABA–glutamine balance, with particular effects on the medial prefrontal cortex [32,64,65].

## 7. Risk Factors for Conversion to Schizophrenia

The potential for SIP to progress into a chronic psychotic disorder is well-documented. In a prior step, socioeconomic status, urban living, history of trauma, and adverse childhood experiences, among others, appear to play a role in increasing vulnerability to substance use disorder [12,66,67].

When it comes to SIP, conversion rates vary based on the substance involved and patient demographics, as well as underlying biological and psychosocial factors. Recent studies suggest an average transition rate of a third of SIP diagnoses converting to schizophrenia [68]. Cannabis is the most strongly associated with subsequent schizophrenia. Repeated or high-potency cannabis exposure, particularly during adolescence, appears to precipitate psychosis in genetically vulnerable individuals, likely due to dysregulation of cortical dopamine transmission and the impairment of neurodevelopmental pruning processes [69,70]. Amphetamine and cocaine use also carry substantial risk, while alcohol-induced psychosis has lower conversion rates, although chronic alcohol use contributes to cognitive decline and neurostructural damage that can complicate long-term recovery [6].

Several risk factors have been identified:

- Early age at onset: Younger individuals are more likely to convert [71], possibly due to neurodevelopmental vulnerability. This vulnerability may reflect incomplete cortical maturation and heightened sensitivity of dopaminergic and glutamatergic systems during neurodevelopment.
- Male gender: Men are overrepresented in both SIP [71] and schizophrenia populations. Possible mechanisms include higher rates of early substance use, increased exposure to potent psychoactive substances, and gender-related differences in stress reactivity.
- Repeated episodes of SIP [71]: Each episode may increase the likelihood of permanent neurochemical or structural brain changes, lowering the threshold for future psychosis.
- Cannabis use in the context of first episode psychosis [68]: Cannabis consumption in the context of an initial psychotic episode markedly increases the risk of persistence and recurrence of psychotic symptoms.
- Length of hospitalization is greater than 7 days following 1st episode psychosis, potentially reflecting greater symptom severity or delayed remission [68].

Beyond these clinical variables, emerging evidence points to a broader network of vulnerability factors, such as:

- Family history or genetic predisposition to schizophrenia or bipolar disorder, often mediated by shared polygenic risk [72,73].
- Childhood adversity and trauma exposure, which amplify dopaminergic reactivity to stress and substances [74,75,76].
- Polysubstance use, which may exert synergistic psychotomimetic effects [77].
- Cognitive deficits and negative symptoms during or after SIP episodes, which represent prodromal schizophrenia profiles [78].
- Poor premorbid adjustment, social isolation, and inadequate treatment adherence, all of which worsen the clinical trajectory [79,80].

Identification of these risk factors has major implications for early intervention. Evidence suggests that early identification and intervention in high-risk individuals can mitigate this risk. Recommended strategies include targeted psychosocial interventions (e.g., cognitive-behavioral therapy, psychoeducation), close clinical monitoring, and supportive case management to address substance use and psychiatric symptoms. In selected cases, carefully considered pharmacological interventions may be indicated to prevent symptom escalation, although these require individualized risk–benefit assessment. Integrating these approaches into early-intervention programs is crucial to reduce the likelihood of progression to chronic psychotic disorders and to improve long-term functional outcomes [81,82].

## 8. Prognosis

Prognosis in SIP is highly heterogeneous and influenced by a complex interplay of biological, psychological, and environmental factors. While many individuals experience complete remission of psychotic symptoms following sustained abstinence and appropriate treatment, a substantial subset develop persistent psychiatric morbidity or eventually transition to a chronic psychotic disorder. In the short term, SIP episodes are often self-limited, particularly when triggered by acute intoxication in individuals without prior psychiatric history. With abstinence, symptoms typically resolve within days to weeks, and full functional recovery is achievable. Outcomes depend largely on the presence and intensity of the risk factors outlined above. Generally, single, brief episodes associated with isolated substance use, absence of a family history of psychosis or mood disorders, in the context of otherwise good premorbid functioning and psychosocial stability, have a favorable prognosis. Early engagement with treatment and rehabilitation services may also be important. Conversely, the prognosis is poorer in individuals with recurrent episodes, chronic substance use, or coexisting vulnerabilities (e.g., family predisposition, previous psychiatric history, trauma exposure) [7,83,84,85]. In these populations, repeated intoxication and withdrawal cycles can precipitate long-term neuroadaptive changes in dopaminergic and glutamatergic pathways, increasing the likelihood of symptom persistence or relapse, resulting in functional deterioration and social decline.

Some studies suggest that SIP may function as an early warning sign or prodrome of schizophrenia [78]. In these cases, the acute psychotic episode represents not merely a transient reaction to a drug but rather the unmasking of a latent psychotic disorder through substance-induced neurochemical stress. In others, persistent use leads to long-term neurobiological changes in brain function, making sustained remission unlikely. Mortality rates are elevated in SIP populations, driven by suicide [39,86,87], accidental overdose, and the physical health consequences of substance use (e.g., hepatic, cardiovascular, and infectious diseases). For example, a longitudinal Finnish cohort demonstrated that individuals with alcohol-induced psychotic disorder (AIPD) exhibited a 20-fold higher mortality compared to the general population and a 12-fold higher rate compared to individuals with alcohol dependence without psychosis over an eight-year follow-up period [12].

Another prognostic challenge arises from poor engagement with care. Many patients show limited insight, fluctuating motivation for treatment, and inadequate social support, leading to discontinuation of follow-up and suboptimal adherence to pharmacological or psychosocial interventions. Sustained engagement with integrated dual-diagnosis programs, combining psychiatric and addiction care, is therefore crucial for improving outcomes and reducing relapse or conversion risk.

Finally, it is important to recognize that cultural, legal, and healthcare system differences across countries may substantially influence the presentation, diagnosis, and management of substance-induced psychosis. Cultural attitudes toward substance use can shape symptom reporting, help-seeking behavior, and social support. Legal frameworks and drug policies affect the availability of substances, patterns of use, and risk of criminalization, while variations in healthcare infrastructure determine access to integrated mental health and addiction services. In countries with legalized or socially accepted cannabis use, increased availability and use of high-potency products may alter the prevalence and clinical features of cannabis-induced psychosis specifically, limiting the generalizability of findings across settings and highlighting the need for cross-cultural research to develop context-sensitive approaches to assessment and long-term management [88].

In summary, while a subset of patients with SIP achieves full recovery, the long-term trajectory in many cases is characterized by partial remission, relapse vulnerability, and an elevated risk of chronic psychosis or mortality. Prognostic precision requires longitudinal assessment, ongoing abstinence confirmation, and early intervention, targeting both psychiatric symptoms and substance use behaviors.

## 9. Treatment and Long-Term Management

The treatment of patients with co-occurring psychotic disorders and substance use disorders depends on the complexity of the interaction between substance use and psychopathology, which generates specific clinical challenges and needs. A crucial element of care is a comprehensive, individualized approach that simultaneously addresses mental and physical health as well as social needs. An important goal is to avoid the exclusion of these patients from mental health services due to substance use [89]. It should also be noted that acute substance-induced psychotic states may require involuntary treatment in emergency settings when there is imminent risk to the patient or others. While ethically justifiable under beneficence and harm prevention, compulsory care raises concerns regarding autonomy, proportionality, and diagnostic uncertainty between substance-induced and primary psychosis. Limited collateral information and intoxication-related behavioral disturbances may increase the risk of inappropriate use of coercive measures. Consequently, involuntary interventions should be time-limited, focused on short-term stabilization, and followed by reassessment. Effective treatment of the underlying substance use disorder depends on voluntary engagement, patient motivation, and therapeutic alliance, emphasizing the need to transition to consent-based interventions once acute symptoms are resolved [90].

Therapeutic approaches typically combine pharmacological and psychosocial interventions and require close collaboration among psychiatrists, psychologists, social workers, and other specialists. Patients with co-occurring disorders face numerous difficulties regarding detection and assessment, access to treatment, and coordination of the necessary services [91]. U.S. data from 2011 indicate that 44% of patients with dual diagnoses receive treatment for one of the two disorders, while only 7% receive simultaneous treatment for both [92]. Although several therapeutic strategies have been developed, research has not yet yielded definitive conclusions about which specific approaches are most effective.

Initial management of substance-induced psychosis (SIP) focuses on acute symptom stabilization, ensuring patient safety, and initiating abstinence from the implicated substance. In most cases, short-term antipsychotic treatment—particularly with second-generation agents such as olanzapine or risperidone—is effective for managing acute symptoms. Agitation may be treated with benzodiazepines. In cases involving psychedelics or hallucinogens, minimal intervention beyond monitoring may be sufficient. Long-term management requires comprehensive care addressing both psychosis and the underlying substance use disorder. Psychotherapeutic interventions such as cognitive-behavioral therapy (CBT), motivational interviewing (MI), and contingency management have demonstrated efficacy in supporting abstinence and preventing relapses. Integration of addiction and psychiatric services is essential. Dual-diagnosis programs providing coordinated, multidisciplinary support improve outcomes and reduce relapses and hospitalization. Follow-up care should continue for one to two years, with regular monitoring of symptom recurrence, substance use, medication adherence, and functional outcomes.

### 9.1. Therapeutic Models of Comorbidity

Treatment models are generally classified into three categories: sequential, parallel, and integrated. In the sequential model, one disorder is treated first, and the second is addressed only once the first has stabilized. Mental health and substance use services function independently, with referrals serving as the primary link between them [93,94]. In the parallel model, both disorders are treated simultaneously but in separate therapeutic settings. Although the two services may interact, each retains therapeutic autonomy and its own approach [95]. Both approaches have significant limitations, particularly due to poor communication and coordination between services, the absence of unified strategies, and high treatment dropout rates [93].

The integrated treatment model involves a coherent plan that addresses both the psychotic and the substance use disorder simultaneously within the same interdisciplinary team. This treatment is typically provided in an outpatient setting. The goal of this model is twofold: to improve access to care for individuals with dual diagnoses and to ensure that pharmacological and psychosocial interventions are effectively combined [96,97]. There is substantial evidence that integrated treatment is the most effective option [98,99,100], even though there are many difficulties and challenges in overcoming the traditional separation between mental healthcare systems and addiction treatment facilities [101]. Data from the NH Dual Diagnosis Study, an earlier longitudinal study of people with schizophrenia and substance use in rural New Hampshire, showed that a high percentage of people who received integrated treatment showed improvement in both their clinical status and their functioning over a 10-year period [102]. Another 7-year prospective study of patients with schizophrenia and substance use disorders from the United States showed that participants showed overall significant improvements in their psychopathology, physical functioning, employment, and life satisfaction. However, the results showed that there were significant variations, meaning that not all patients experienced the same level of benefit [103].

Integrated treatment approaches are based on several principles: 1. Working heavily on a harm reduction framework; 2. Implementation of motivationally oriented therapeutic interventions, recognizing varying levels of readiness for change and using techniques to identify and instill their desire to engage in treatment and make positive changes; 3. Implementation of cognitive-behavioral techniques to foster adaptive coping strategies and challenge maladaptive beliefs; 4. Focus on improving overall functioning, including occupational performance, daily activities, and social relationships; 5. Activation of the individual’s social support system, including involvement of family members, friends, and other significant persons, which is a critical factor for positive outcomes [104].

Multiple studies, primarily from the United States, show that most individuals with mental disorders and co-occurring substance use disorders do not receive treatment for their substance use problem [105]. Even among those who do receive care, treatment often addresses only mental health needs. Approximately one-third of individuals with co-occurring opioid use disorder and a serious mental disorder report receiving treatment for both conditions within the past year—a proportion notably higher than among those with mild or moderate mental disorders [106]. Treatment discontinuation is a major challenge in this population; a recent review reported an average dropout rate of nearly one-third (27.2%) [107]. Therefore, interventions that focus on enhancing engagement in treatment and especially on creating a strong therapeutic relationship are essential. Motivation to discontinue substance use is often low, and recovery may require months or years. Harm reduction is therefore a crucial component, especially for individuals who are not ready to pursue abstinence [104]. Supporting patients in creating a meaningful life not centered around substance use is fundamental. Exploring personal goals and aspirations can facilitate desired changes, even when individuals are not yet prepared to directly address their substance use [104]. Involving family members and other significant people in the treatment process is also vital for supporting recovery [108].

### 9.2. Psychosocial Interventions for Co-Occurring Disorders

Several psychotherapeutic approaches have been employed in the treatment of mental disorders co-occurring with addictions. These include contingency management (CM), cognitive-behavioral therapy (CBT) and relapse prevention, motivational interviewing (MI), combined CBT and MI, brief MI-based interventions, family interventions, and assertive community treatment (ACT) [109]. All these approaches demonstrate low-to-moderate levels of evidence and should be implemented when appropriate training and expertise are available.

Brief Motivational Enhancement/Skills Training: This therapeutic approach helps individuals overcome ambivalence and resistance, enhance self-efficacy related to substance use, and acquire the skills needed to reduce or stop use. Matching interventions to an individual’s stage of motivation and instilling hope for change are central components [110,111,112].

Cognitive Behavioral Therapy (CBT): Cognitive Behavioral Therapy encompasses a range of techniques aimed at developing more effective skills for managing psychiatric symptoms, emotional distress, and interpersonal difficulties. Key techniques for co-occurring disorders include social skills training, training in managing painful emotions and thoughts, development of healthy leisure activities, and cognitive restructuring [104,113].

A review of 32 studies involving more than 3000 patients with serious mental illnesses and co-occurring substance use disorders found no conclusive evidence that any single psychosocial intervention is superior in improving treatment retention or reducing substance use or psychotic symptoms. These studies evaluated diverse interventions—including CBT, MI, skills training, and integrated models of care. Some evidence suggests modest benefits of motivational interviewing, whether alone or combined with CBT, for reducing substance use [109]. However, methodological heterogeneity, variation in outcome measures, and high dropout rates limit the strength of current conclusions. Higher-quality trials are needed to better assess the effectiveness of psychosocial treatments [109].

### 9.3. Pharmacological Treatment for Co-Occurring Disorders

Antipsychotic medications are widely recognized as the mainstay of treatment for individuals presenting with psychosis and comorbid substance use disorders, particularly for reducing psychotic symptoms. Despite the proven efficacy of haloperidol in treating psychotic symptoms, second-generation antipsychotics (e.g., risperidone, olanzapine, and ziprasidone) are generally preferred due to better tolerability and a lower risk of extrapyramidal side effects (Grade: Low) [97,109,114,115,116]. Some evidence suggests that long-acting injectable (LAI) second-generation antipsychotics may be more effective than first-generation LAIs, though conclusive data are lacking [92]. A review of 19 randomized clinical trials found that (a) clozapine was more effective than other antipsychotics in reducing substance use, (b) risperidone was more effective than olanzapine in reducing cravings, and (c) olanzapine, clozapine, and risperidone were superior in improving psychotic symptoms [117].

An Italian survey of prescribing practices for psychosis with co-occurring substance use disorders found that aripiprazole and olanzapine were the most prescribed medications for both acute and maintenance treatment. About half of clinicians also used LAI antipsychotics, with paliperidone being the preferred option. Open-label randomized trials further support the comparative effectiveness of olanzapine, clozapine, and risperidone in reducing substance use and increasing abstinent days [118]. Concurrent substance use can reduce treatment adherence and interfere with antipsychotic efficacy, exacerbating symptoms or affecting pharmacodynamics. Conversely, worsening psychiatric symptoms can increase the risk of substance use. LAI antipsychotics may mitigate these challenges by improving adherence and reducing the number of patients classified as treatment resistant [118]. Ultimately, prescribing decisions are influenced by clinician experience, national health system guidelines, and general schizophrenia treatment guidelines.

Long-term management of psychosis with comorbid substance use disorders remains an area with limited and fragmented evidence. Although integrated treatment models are frequently recommended, most available data derive from short-term studies and do not adequately address sustained outcomes such as relapse prevention, functional recovery, and long-term treatment adherence. The literature is further limited by methodological heterogeneity, variable definitions of integrated care, and the underrepresentation of individuals with dual diagnoses in controlled clinical trials. Consequently, substantial gaps persist regarding the optimal components, intensity, and duration of integrated interventions across different stages of illness. Future research should prioritize longitudinal and pragmatic study designs evaluating multidisciplinary, continuous care models that combine pharmacological treatment, psychosocial interventions, and substance use management within real-world settings. Greater emphasis on early intervention, continuity of care, and community-based services is needed to inform evidence-based long-term treatment strategies for this complex clinical population [119,120].

## 10. Limitations

The present study has limitations inherent to the narrative nature of the review. The absence of a predefined systematic protocol may increase the risk of selection bias and limit the reproducibility of the search process. Study selection and interpretation relied partly on the authors’ scientific judgment, which may affect the completeness of the literature coverage. Nevertheless, the narrative approach enables the integrative synthesis of heterogeneous evidence. This facilitates a clinically oriented overview of the complex comorbidity between psychosis and substance use disorders.

## 11. Conclusions

Substance-induced psychosis (SIP) represents a clinically complex phenomenon at the intersection of addictive and psychotic disorders. Although traditionally considered transient, growing evidence demonstrates that SIP frequently signals an underlying vulnerability to persistent psychotic illness, with up to one-third of individuals subsequently converting to schizophrenia. This risk is particularly elevated among young males, those with repeated episodes, prolonged hospitalizations, or exposure to high-risk substances such as cannabis, amphetamines, and cocaine. Neurobiological studies highlight heterogeneous pathophysiological mechanisms—ranging from dopaminergic and glutamatergic dysregulation to disturbances in endocannabinoid signaling—reflecting the diverse pharmacological profiles of the substances involved.

**Figure 1:**
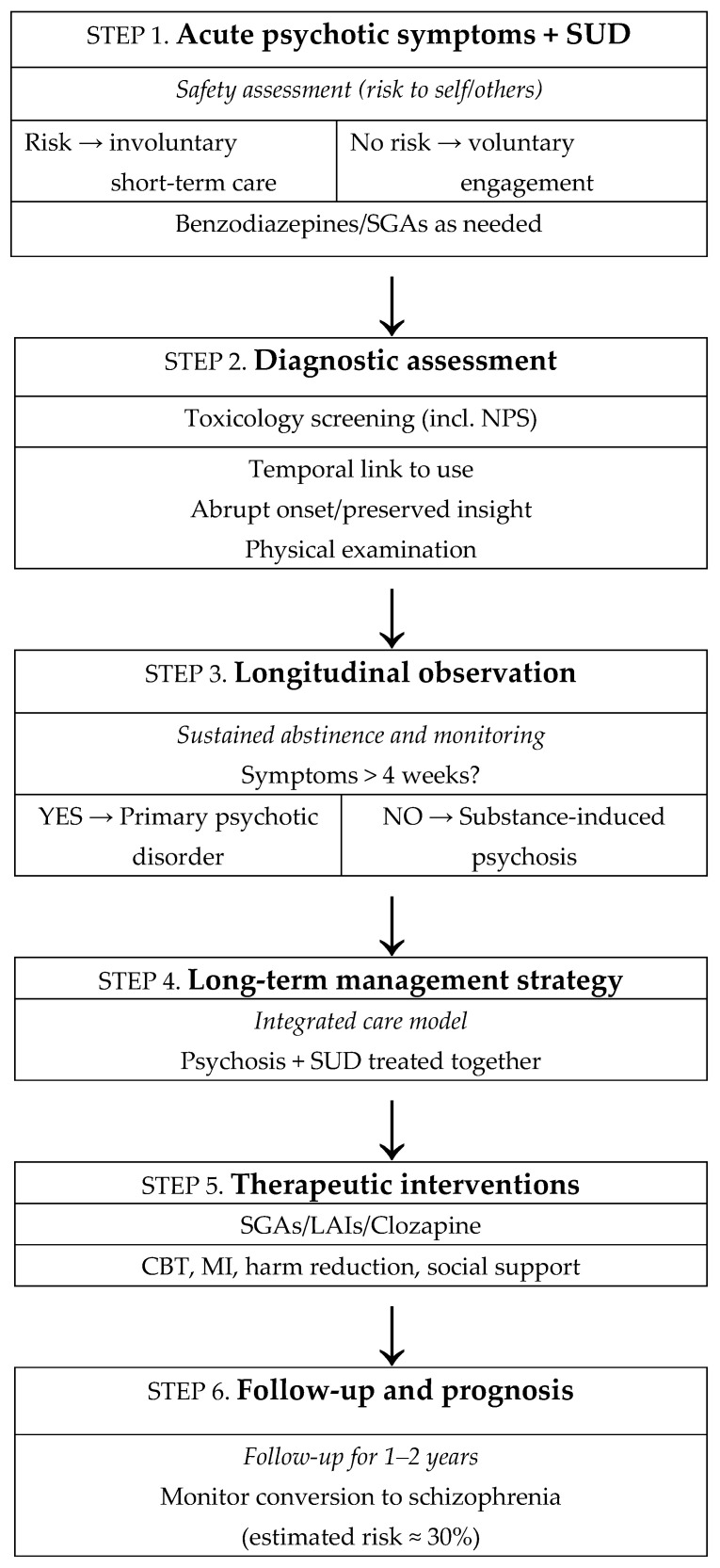
Flowchart: Management of Substance-Induced Psychosis.

Accurate diagnosis requires careful clinical evaluation, temporal assessment of substance use relative to symptom onset, supported by detailed collateral history and appropriate advanced toxicological screening. Differentiating SIP from primary psychosis remains challenging, underscoring the importance of longitudinal monitoring, particularly during sustained abstinence. Therapeutic management must address both psychosis and substance use within an integrated, multidisciplinary framework. Psychosocial interventions offer modest benefits, though high dropout rates and methodological variability limit definitive conclusions. Pharmacologically, second-generation antipsychotics, particularly clozapine, risperidone, and olanzapine, appear beneficial, with long-acting formulations potentially enhancing adherence. Given the widespread use of an ever-increasing variety of psychoactive substances, SIP represents a major public health concern, associated with substantial morbidity and a significant risk of becoming chronic. Early identification, sustained engagement, harm-reduction strategies, and integrated dual-diagnosis treatment are essential to improving long-term outcomes for this vulnerable population (Figure 1). Psychosis comorbid with substance use disorders poses a significant public health challenge, with elevated risks of relapse, functional decline, and premature mortality. Integrated treatment models combining pharmacological, psychosocial, and substance-focused interventions show promise, but evidence for long-term effectiveness remains limited. These findings underscore the urgent need for coordinated mental health and addiction services, ensuring continuity of care, early intervention, and accessible community-based support. Advancing longitudinal research and implementing evidence-based integrated approaches are essential to reduce morbidity, enhance recovery, and improve quality of life for this vulnerable population [121].

## Figures and Tables

**Table 1:** Key neurotransmitters and mechanisms of action of substances that can induce psychosis as well as relevant symptoms.

Substance	Neurotransmitters	Mechanism	Symptoms Frequent in Psychosis Presentation
Amphetamines	DA, NE	Reuptake inhibition and intracellular cytotoxicity	paranoia, visual hallucinations, formication
Synthetic Cathinones	5HT, DA	Reuptake Inhibition	psychomotor agitation
PCP/Ketamine	DA, Glutamate	Partial D2 agonist, NMDA antagonism	positive and negative symptoms, behavioral disturbance
Cocaine	DA	Reuptake inhibition	paranoia, visual hallucinations, formication
LSD	5HT	5HT2 auto-receptor agonist	visual distortions, dissociation
Psilocybin	5HT	5HT1, 5HT2 auto-receptor agonist	visual distortions, dissociation
Cannabis	Cannabinoids	Endocannabinoid receptor agonists (CRB1-R)	paranoia, occasionally negative symptoms
Synthetic Cannabinoids	Cannabinoids	Endocannabinoid receptor agonists at higher affinities than THC excluding cannabidiol	agitation, confusion, bizarre behavior
Alcohol	GABA, Gln/GLU	Increased GABA, Gln in Cingulate, decreased GABA in Nucleus Accumbens	visual and haptic hallucinations, AIPD occurs in clear sensorium

DA: dopamine; 5HT: serotonin; GABA: γ-aminobutyric acid; Gln: glutamine; GLU: glutamate; NE: norepinephrine; NMDA: N-methyl-D-aspartate.

## Data Availability

The data presented in this study were retrieved through literature search using the strategy described in the Section 2.
